# Evaluation of the Preschool Situational Self-Regulation Toolkit (PRSIST) Program for Supporting children’s early self-regulation development: study protocol for a cluster randomized controlled trial

**DOI:** 10.1186/s13063-018-2455-4

**Published:** 2018-01-24

**Authors:** Steven J. Howard, Elena Vasseleu, Cathrine Neilsen-Hewett, Ken Cliff

**Affiliations:** 1 Early Start, University of Wollongong, Wollongong, NSW 2522 Australia; 2 School of Education, University of Wollongong, Northfields Avenue, Wollongong, NSW Australia; 3 School of Psychology, University of Wollongong, Wollongong, NSW 2522 Australia

**Keywords:** Self-regulation, Preschool, Professional development, Formative assessment, Child activities, Adult practices, Pedagogy

## Abstract

**Background:**

For children with low self-regulation in the preschool years, the likelihood of poorer intellectual, health, wealth and anti-social outcomes in adulthood is overwhelming. Yet this knowledge has not yielded a framework for understanding self-regulatory change, nor generated particularly successful methods for enacting this change. Reconciling insights from cross-disciplinary theory, research and practice, this study seeks to implement a newly developed program of low-cost and routine practices and activities for supporting early self-regulatory development within preschool contexts and to evaluate its effect on children’s self-regulation, executive function and school readiness; and educator perceived knowledge, attitudes and self-efficacy related to self-regulation.

**Methods/design:**

The Early Start to Self-Regulation study is a cluster randomized, controlled trial for evaluating benefits of the Preschool Situational Self-Regulation Toolkit (PRSIST) program, when implemented by early childhood educators, compared with routine practice. The PRSIST program combines professional learning, adult practices, child activities and connections to the home to support children’s self-regulation development. Fifty preschool centers in New South Wales, Australia, will be selected to ensure a range of characteristics, namely: National Quality Standards (NQS) ratings, geographic location and socioeconomic status. After collection of baseline child and educator data, participating centers will then be randomly allocated to one of two groups, stratified by NQS rating: (1) an intervention group (25 centers) that will implement the PRSIST program; or (2) a control group (25 centers) that will continue to engage in practice as usual. Primary outcomes at the child level will be two measures of self-regulation: Head-Toes-Knees-Shoulders task and the PRSIST observational assessment. Secondary outcomes at the child level will be adult-reported measures of child self-regulation, executive function and school readiness. Outcomes at the educator level will involve a survey of their perceived knowledge, attitudes and self-efficacy for supporting children’s self-regulatory development. In all cases, data collectors will be blinded to group allocation.

**Discussion:**

This is the first randomized controlled trial of a new program to foster early self-regulation, using low-cost practices and activities that are aligned with early-years contexts, routines and practices. Results will provide important information about the efficacy of this approach and evaluate its underlying model of self-regulatory change.

**Trial registration:**

Australian New Zealand Clinical Trials Registry, ACTRN12617001568303. Registered on 21 November 2017.

**Electronic supplementary material:**

The online version of this article (10.1186/s13063-018-2455-4) contains supplementary material, which is available to authorized users.

## Background

There is evidence that early self-regulation abilities robustly predict long-term trajectories of health, wealth and criminality into adulthood [1]. For children with low self-regulation in the preschool years, the likelihood of poorer academic outcomes in school [2, 3] and poor physical health, substance abuse, financial difficulties and criminal offending in adulthood is overwhelming [1, 4, 5]. Is this mere association? The fact that these effects persist even after accounting for individual differences in intelligence, socioeconomic status and home learning environments establishes self-regulation as a critical link in a probable causal sequence, and a potential target for intervention [1].

Research has further shown self-regulation to be malleable, with children who become more self-controlled achieving better outcomes in adulthood [1]. Early interventions, in particular, have been suggested to produce more pronounced, stable and lasting change [6], and are more likely to produce a greater return on financial investment [7]. Moreover, long-term benefits of self-regulation intervention can be expected not only for children with low self-regulation in preschool, but also amongst those who have average or above average self-regulation abilities [1]. Interventions fostering early self-regulation therefore represent a promising opportunity to improve individuals’ lifespan trajectories and, as a consequence, reduce the societal and financial burdens of negative health, wealth and anti-social outcomes. Despite these compelling findings, this knowledge has not yet yielded a theoretical framework for understanding self-regulatory change, nor has it generated overly successful and consistent methods for enacting this change.

### Existing interventions for fostering self-regulation

Self-regulation remains an ill-defined construct, characterized by multiple independent lines of inquiry. One line of self-regulation research derives from a landmark paper in “Science” [8] that reported 4-year-olds’ performance on a single delay-of-gratification task, in which they had to resist eating a marshmallow in order to receive an enhanced reward. Performance on this task robustly predicted self-regulatory outcomes into adulthood. In that paper Mischel and colleagues [8] speculated that cognitive and attentional control processes were an essential feature of self-regulation. Much cognitive psychology and neuroscience research that followed focused on executive functions (EFs) as a core component of self-regulation. More recently, this research tradition is manifest in a proliferation of computerised “brain training” programs that target EFs as a means of promoting self-regulatory abilities – an industry now worth more than one billion dollars [9]. In this context, brain training refers to a collection of programs in which users engage in activities at an increasing level of challenge to promote more effective higher-order cognitive functioning. These time-intensive and cost-intensive programs, however, often yield only modest gains and limited transfer of benefits to untrained tasks, domains and real-world outcomes [10]. Further, these programs are non-routine – children have to be removed for individual supervised sessions – and are not well-suited to fostering the self-regulatory development of young children within their social context [11].

In contrast, Carver and Scheier’s influential feedback loop model of self-regulation [12] proposes that successful self-regulation is a function of a continual process of “test-operate-test-exit” (TOTE). In the test phase, one’s current state is compared against the desired goal state. When these are discrepant, actions (“operations”) are undertaken to reduce the discrepancy and then the cycle repeats. On the basis of this approach, Baumeister and Heatherton identified three essential components necessary for successful self-regulation: selection of a goal; motivation to achieve the goal by reducing the discrepancy between current and goal states; and sufficient capacity to achieve this goal [13]. In line with this theoretical model, educational and social psychological research has tended to focus on the behavioural, social and emotional features of self-regulation, such as persistence in difficult tasks, impulsivity, frequency of temper tantrums and so forth [13, 14]. Interventions in this self-regulation tradition have therefore tended to target children’s goal setting and motivation through classroom activity and curricular approaches, with similarly mixed results [10, 15, 16].

Although the TOTE model offers a possible explanation for (un)successful self-regulatory behaviours, it neglects the potential role of cognitive control processes (i.e., EFs) and does not make clear what mechanisms permit the effortful execution of self-regulation. Currently there have been few attempts to reconcile these separate lines of investigation [14]. As such, an integrative model for understanding self-regulatory change is needed.

### Proposed models of self-regulatory change

In response, Hofmann et al. proposed that EFs may represent the capacity component of self-regulation [14]. In this modification, EFs provide the ability to control attention and remain goal-directed despite competing stimuli and interests. Successful self-regulation, according to this revision, also requires that a goal to self-regulate has been adopted and that there is a sufficient level of motivation for continuing to pursue this goal. Although it has not yet been empirically evaluated, this integrative model may explain why many existing interventions show limited transfer to real-world outcomes. That is, EF interventions may be limited because they improve only the capacity component of self-regulation. Similarly, existing self-regulation interventions likely do not address the cognitive control processes that contribute to successful self-regulation. If supported, such a model would enhance our understanding of the factors that contribute to successful self-regulation and self-regulatory failures, and the need to target each of these factors to enhance young children’s self-regulation abilities.

From this proposition a self-regulation intervention approach emerges that acknowledges the cognitive and socially mediated mechanisms of self-regulatory change. Departing from the growing “brain training” tradition, this approach investigates current routine practices that engage and extend (or provide an opportunity to engage and extend) young children’s ability to self-regulate. Specifically, early research by Howard and colleagues [17] in this area has established benefits from embedding cognitive and self-regulatory challenge in the context of shared book reading with preschoolers. This research suggests the feasibility of achieving EF and self-regulation benefits from low-cost to no-cost everyday activities, thereby expanding the range of settings, contexts and activities available for fostering self-regulatory abilities (e.g., books, games, play, groups, outdoor spaces).

This project thus seeks to implement and evaluate a broader program of professional learning, adult practices, child activities and home connections that can be readily integrated into preschool contexts and implemented by early-years educators. This Preschool Situational Self-Regulation Toolkit (PRSIST) Program aims to engage, challenge and extend children’s self-regulation in ways that are play-like and target all of the aspects required for successful self-regulation (i.e., goal setting, motivation, problem solving, self-regulatory capacity). Efficacy of this intervention will be evaluated in terms of improvements in children’s self-regulatory development (and related abilities, such as EFs and school readiness), and educators’ reports of their knowledge, attitudes and self-efficacy related to supporting children’s self-regulation. It is expected that the intervention will have a positive effect on these identified child and educator outcomes.

## Methods/design

### Study design

**Fig. 1:**
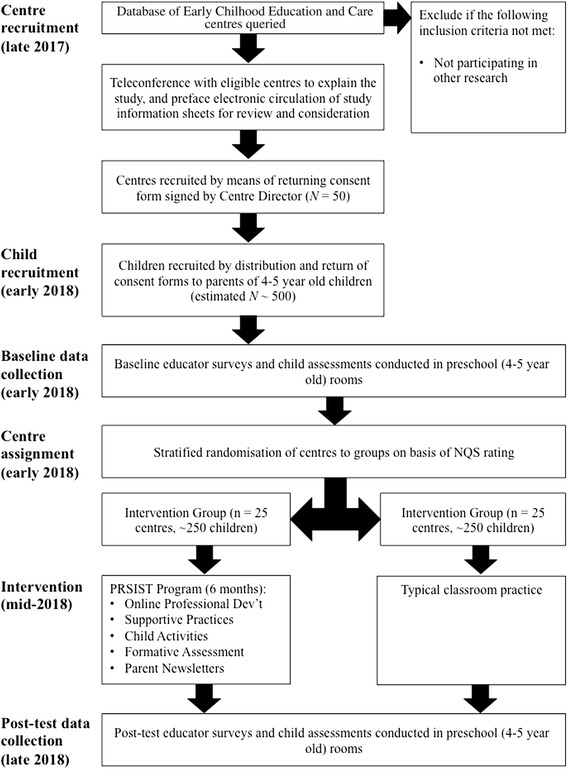
Flow diagram of the stages of the PRSIST Program evaluation

**Fig 2:**
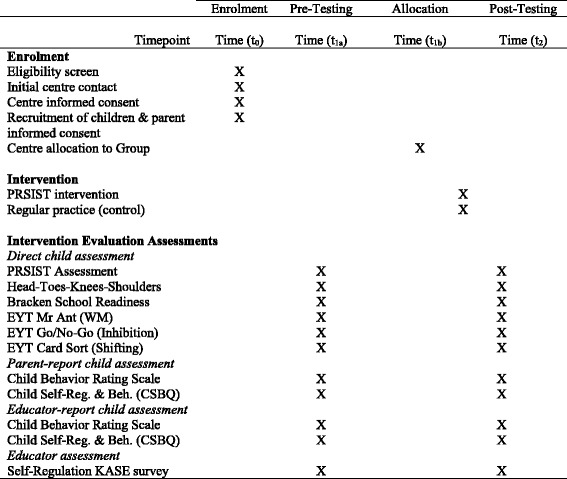
SPIRIT figure

The study employs a clustered, randomized controlled, trial design. Fifty preschool centers in New South Wales (NSW), Australia, will be recruited to ensure representation of NQS ratings (Working Towards, Meeting, Exceeding), location (metro, regional), and socioeconomic areas (as based on the Socio-Economic Indexes for Australia; SEIFA). The sample will ensure representation across these variables, but is not intended to be fully representative of NSW preschool services. After collection of baseline child and educator data, participating centers will be stratified by their pre-existing NQS rating and randomly allocated to one of two groups: (1) an intervention group (n = 25 centers) receiving the professional development (PD) intervention; or (2) a control group (n = 25 centers) that will continue engaging in practice as usual. Educators in the intervention group will implement the PRSIST Program for 6 months from April 2018. At the end of the intervention (from October 2018), fieldworkers, blinded to group allocation, will conduct post-intervention child assessments and educators will again complete the self-regulation survey. A flowchart depicting the sequence of recruitment, intervention and assessment in this study is shown in Fig. 1. An outline of Standard Protocol Items: Recommendations for Interventional Trials (SPIRIT) time points and actions is shown in Fig. 2 (see Additional file 1).

### Preschool center characteristics and recruitment

Criteria for the potential inclusion of centers will be that centers are not participating in other research. Centers will be invited to participate in the study first by an initial call or email to gauge interest. A teleconference call to brief interested centers on the nature and requirements of the study will then be established and offered. This will be followed by email circulation of Director information sheets and consent forms to be reviewed and, for centers willing to participate, signed and returned. Participating centers will be those that meet the inclusion criteria and return a signed Director consent form to participate in the study. The first 50 preschool centers that provide written consent will take part in the study.

### Child characteristics and recruitment

Following center recruitment, and preceding the intervention, children in the final preschool year (4–5 years of age) will be recruited from participating centers. This involves circulating information sheets and consent forms, via the center, to parents or legal guardians of suitably aged children. Participating children will be those that meet the age-inclusion criteria, return a signed parental consent form and provide verbal assent to participate. Fifty preschool centers are expected to yield a sample of approximately 500 children ages 4–5 years, with whom the child assessments will be conducted. There are no further exclusion criteria for child participation.

### Randomized allocation of centers

After baseline data collection is completed, all participants (i.e., educators and children) will be assigned to control or intervention groups randomly by cluster (center) using a computer-generated random number sequence. Randomization will not occur until after: (a) recruitment of centers is complete; and (b) initial baseline data collection is complete. As such, those involved in recruitment of centers will be unaware, at the time of recruitment, to which group centers will be allocated. Stratified randomization of centers (cluster) will be conducted using NQS rating as the core stratification variable.

### Outcome measures and procedures

All measures will be administered at baseline and again after the 6-month intervention period (post-test). The battery of child measures was selected to include multi-source measures of children’s self-regulation (objective, observational, educator-report, parent-report) and related abilities. In total, the child measures involve approximately 60 minutes of direct assessment per child (split into three sessions of 20 minutes each) and around an hour of educator time per center (5 minutes of educator time per child for the educator-report self-regulation measure, and 15 minutes to complete a survey of educators’ perceived self-regulation knowledge, attitudes and self-efficacy). In all cases, a trained fieldworker will carry out child assessments in a quiet area of the child’s preschool center. In all cases, fieldworkers collecting the baseline and outcome data will be blinded to each center’s group allocation.

Primary child-level outcomes consist of two measures of children’s self-regulation, namely the Head-Toes-Knees-Shoulders (HTKS) assessment [18] and PRSIST assessment [Howard, Neilsen-Hewett & de Rosnay: An observation assessment of early self-regulation: validity and reliability of the Preschool Situational Self-Regulation Toolkit (PRSIST) Assessment, in preparation]. HTKS, which takes around 7 minutes to administer, is an objective measure of child self-regulation (κ = 0.90 for inter-rater reliability; α > .90 for internal consistency; *r*s from .29 to .53 for concurrent validity with executive function measures,) [19] that requires children to perform a different action to that indicated by the administrator (e.g., touch their knees when the facilitator says “touch your head”). The PRSIST assessment, which takes about 15 minutes to administer, is an 11-item observational measure of children’s self-regulation (showing strong concurrent validity with other self-regulation and related measures) that involves completing a structured observation scale on a child’s performance within a specified activity (e.g., a memory-card-matching game in a group of four children). Each item, rated on a 7-point scale, provides an elaboration of the item’s meaning and example descriptions of performance at its extremes. This scale yields subscales related to cognitive self-regulation (e.g., “Did the child sustain attention, and resist distraction, throughout the instructions and activity?”), behavioural self-regulation (e.g., “Did the child control their behaviours and stay within the rules of the activity?”) and social-emotional self-regulation (e.g., “Did the child recover from being upset, disappointed or frustrated?”) Both scales require completion of an online training module to ensure reliability prior to conducting the assessments in field.

Secondary outcomes at the child level are: parent-report and educator-report measures of child self-regulation, namely the 10-item Child Behavior Rating Scale (CBRS; showing strong validity and reliability in previous studies) [20] and the 33-item Child Self-Regulation and Behaviour Questionnaire (CSBQ; α > .80 for internal consistency of self-regulation subscales; *r*s > .60 for concurrent validity with a similar questionnaire measure) [11]; three iPad-based measures of executive function from the Early Years Toolbox (EYT), namely working memory (Mr Ant;, *r* = .46 for concurrent validity with comparison working memory measure), inhibition (Go/No-Go; internal consistency, α > .80; concurrent validity with comparison for inhibition measure, *r* = .40) and shifting (Card Sort; *r* = .45 for concurrent validity with comparison working memory measure) [11]; and the Bracken School Readiness Assessment (BSRA; split-half reliability, *r* = .95; *r*s > .60 for concurrent validity with language impairment measure) [21].

Educator-level outcomes are measured by the Self-Regulation Knowledge, Attitudes and Self-Efficacy (KASE) survey. Based on Bandura’s [22] self-efficacy scales, each item asks the educator to rate their perceived knowledge of self-regulation, attitudes about children’s self-regulation, or self-efficacy for fostering children’s self-regulation on a scale from 0 (no knowledge, do not agree at all, or cannot do) to 100 (know everything there is to know, fully agree, very certain can do). This format, phrasing and scale of items have been shown to have good reliability and validity [22]. At post-intervention assessment, educators will be provided with their initial ratings for perceived knowledge and asked to: (1) modify their initial ratings, if necessary; and then (2) indicate their current ratings. This will permit potential upward or downward revision of initial estimates of their knowledge, which could be a consequence of undertaking the PRSIST Program (e.g., later recognizing what was not fully understood, and then working to remedy this gap in knowledge).

### Preschool Situational Self-Regulation Toolkit (PRSIST) Program intervention

The PRSIST Program aims to engage, challenge and extend young children’s self-regulation in ways that are playful, low-cost, routine, and target all the aspects required for successful self-regulation (i.e., goal setting, motivation, problem solving, self-regulatory capacity). The PRSIST Program is a collection of professional learning, adult practices, low-cost, play-based and everyday child activities, and home connections to support the development of children’s self-regulation. The PRSIST program has been designed to be compatible across a range of early-learning contexts, but in the current study will be implemented by preschool educators. In previous phases of this research, all program elements were piloted, evaluated and revised on the basis of feedback from early-years educators (e.g., child and educator enjoyment, program compatibility with preschool contexts, routines and practices, perceived benefit). In line with this feedback, the program has also been developed so that it can be implemented for varying durations, intensities, and using different combinations and sequences of elements. However, for the current trial educators will implement the program over the course of 6 months. Over this period, all participating centers will implement each of the program’s 4 core elements:

1. Adult practices: educators will be provided with a practice manual that describes eleven principles, and associated practices, to support children’s self-regulation development and minimise factors that undermine self-regulation (e.g., stress, sadness). In the manual principles are described (e.g., foster intrinsic motivation through use of encouragement), contextualized in a real-life scenario to illustrate its importance (e.g., a child shows an educator a construction they have worked hard on), and specific practices are provided related to the principle (e.g., open-ended questioning). These principles and practices are further supported by nine online professional development videos that expand on these principles and practices (available on the program website (www.prsist.com.au)), which educators will be asked to engage with in the first 2 months of the program.
2. Child activities: in addition to the adult practices, a collection of 28 play-based activities aims to extend children’s self-regulatory capacity. These activities were developed from practices already occurring in high-quality preschool services; minimal modification of existing practices in high-quality centers (modified to maximise self-regulatory benefit); or newly created activities that have been piloted and revised based on the feedback of educators across a range of preschool services. The programe has been designed so that the timing, intensity, selection and sequence of child activities is flexible; however, within the current trial educators will be asked to complete a minimum of three child activities, of their choosing, in each week of the intervention period. The fidelity of this intensity requirement will be evaluated through monthly wall-calendar sticker charts, returned to the research team, which show the date and frequency each activity was conducted. The activities are each described on the program’s website (www.prsist.com.au), and are also compiled into a series of children's book as an easy entry for educators to read about and conduct the activities (i.e., each children's story has activities linked to central plot points, and then compiles all of the activities in an appendix at the end of the book (see an example in Fig. 3).

**Fig. 3:**
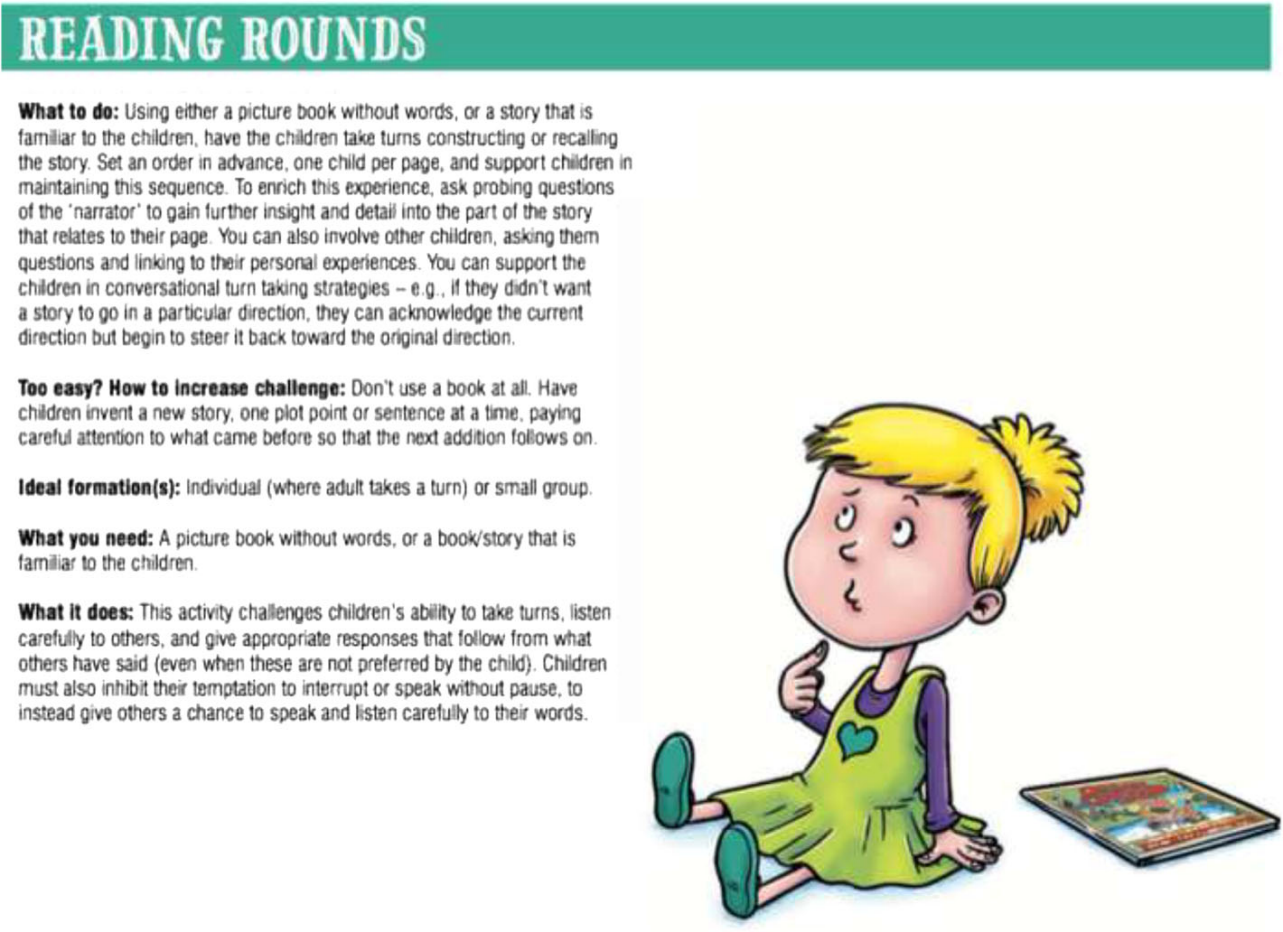
Sample child activity from the appendix of *The Pear That Wasn’t There*, which is focused on social and emotional self-regulation
3. Formative assessment of self-regulation: providing an appropriate environment and experiences to support children’s development requires information about their current developmental progress. To support an understanding of where children are up to in their self-regulation development, participating preschool educators will be given access and online training for our PRSIST formative assessment tool. This tool uses observation of children as they perform everyday activities, but structures this observation to: (1) focus on key areas of self-regulation; and (2) provide actionable data based on a child's current developmental progress. Educators will be able to use this tool (recommended frequency of at least twice per child during the intervention period for the current trial) to tailor the complexity of the child activities to children’s current developmental needs.
4. Parent newsletters: while the program is not specifically being implemented by parents in this trial, monthly parent newsletters will foster important connections with the home. Monthly newsletters for parents, to span the course of the program, inform parents about their child's activities in their preschool center, about self-regulation and its importance, and give suggestions for how parents can additionally support their child's self-regulation at home. Each newsletter consists of one double-sided page, and will be disseminated by the child’s preschool center.

The PRSIST program has been designed to be flexible (in response to the needs of educators and parents working with young children), low cost (so practices and activities can be in a range of contexts and socioeconomic conditions), and freely and widely disseminated (after this evaluation is complete, anyone can access the program website at no cost, to be able to access the program in part or in full). It is noted that given this potential flexibility in design, subsequent research would need to evaluate efficacy in differing contexts, durations and intensities of implementation.

### Statistical analyses and power

The primary outcomes are changes in children’s self-regulation, which will be analyzed in multi-level models where a specific intervention-control comparison will be included. The analyses will be carried out using two different types of dataset: intention-to-treat*,* including all participants according to the random allocation, irrespective of whether the intervention was implemented with fidelity (to estimate program effects if implemented more broadly); and per-protocol, including all participants for which the program was implemented to the minimum requirements described in the “PRSIST Program intervention” section (to estimate program effects if implemented with fidelity).

To evaluate the effect of the PRSIST Program intervention, changes in child outcomes will first be measured in a multi-level model where a specific intervention-control comparison will be included. Based on an ICC of .12 and a small effect size, 500 participants will be sufficient to detect a child-level effect at 80% power, alpha of .05 and 10% attrition. For educator-level data, based on an ICC of .05 and a moderate effect size, 120 participants (at least two educators per center) will be sufficient to detect this effect at 80% power and alpha of .05. These ICCs were anticipated on the basis of previous large-scale research in children clustered within different preschool settings [23], and anticipated effect sizes were based on initial small-scale piloting of this approach to promoting self-regulation [17]. Multiple imputation methods will be used for missing individual outcome data. Covariates to be considered in the analyses include the NQS, child age, child sex, maternal education and socioeconomic status.

### Ethical and research governance approval

The study was granted ethical approval by the University of Wollongong Human Research Ethics Committee Social Sciences (HE2017/451) on 7 November 2017. Written consent will be obtained from Center Directors (for center participation), educators (for participation and completion of educator-report measures), and children’s parent(s) or legal guardian(s) as a condition for participation. This will include consent for publication of the study results in anonymized aggregate format. Per the Consolidated Standards of Reporting Trials (CONSORT) guidelines, the study’s final reporting will follow the CONSORT statement and its relevant extensions (e.g., cluster trials, non-pharmacological interventions).

### Study timeline

Recruitment of centers will commence in January 2018 and recruitment of children will begin in February 2018. Pre-test data will be collected in March and April 2018, with post-test data collected 7 months after pre-test in October and November 2018. Figure 1 provides details of the stages of the study. The trial is set to finish in December 2018.

## Discussion

Success of this evaluation trial is dependent on the cooperation of centers and staff recruited to the study, and their implementation of the PRSIST Program with high fidelity, and the participation of parents and children from those centers. As such, substantial efforts have been expended in producing informative communication materials and easy-to-use program materials. We believe that the necessary groundwork has been laid, and the next publication on this trial will present results of the efficacy of the PRSIST Program for enhancing children’s self-regulation and related abilities, and educators’ perceived knowledge, attitudes and self-efficacy in supporting children’s self-regulation. The results of this study will provide insight into whether self-regulation benefits can be achieved through low-cost and routine practices, when these are done more intentionally (rather than incidentally), with the aim of maximising self-regulatory challenge and benefit, and in a way that is flexible and compatible with current early-years contexts, practices and routines. If efficacious, this would be a significant departure from many current alternative approaches, which can be difficult to implement with fidelity, and are often costly and time consuming.

### Trial Status

Recruitment of preschool centers will begin in December 2017. Recruitment of educators and children will begin in February 2018. Following baseline data collection in March and April 2018, centers will be randomly assigned to groups and the PRSIST Program will commence in the intervention centers. Post-intervention child assessments and educator surveys will be collected in October and November 2018.

## Additional file

Additional file 1: SPIRIT checklist. (DOC 124 kb)

## Abbreviations

ACTRN: Australian New Zealand Clinical Trials Registry
BSRA: Bracken School Readiness Assessment
CBRS: Child Behavior Rating Scale
CSBQ: Child Self-Regulation and Behaviour Questionnaire
EF: Executive function
EYT: Early Years Toolbox
HTKS: Head-Toes-Knees-Shoulders
ICC: Intra-class correlation
KASE: Knowledge, Attitudes and Self-Efficacy
NQS: National Quality Standards
NSW: New South Wales
PD: Professional development
PRSIST: Preschool Situational Self-Regulation Toolkit
TOTE: Test-operate-test-exit
